# An efficient method of modulo adder design for Digital Signal Processing applications

**DOI:** 10.1016/j.mex.2025.103216

**Published:** 2025-02-15

**Authors:** Subodh Kumar Singhal, Sumit Kumar, Sujit Kumar Patel, K. Anjali Rao, Gaurav Saxena

**Affiliations:** a Jaypee University of Engineering and Technology, Guna, M.P, India; b Symbiosis Institute of Technology, Pune Campus, Symbiosis International (Deemed University), Pune, 412115, Maharashtra, India; c Thapar Institute of Engineering and Technology Patiala, Punjab, India; d Indian Institute of Information Technology Allahabad, Allahabad 211015, India

**Keywords:** RNS, Parallel prefix adder, VLSI, Modulo adder, Diminished-1 modulo adder

## Abstract

Modulo adder is a widely used arithmetic component in many Digital Signal Processing (DSP) applications such as Finite Impulse Response (FIR), Infinite Impulse Response (IIR) filters, digital signal processors, image processing modules, discrete cosine transform, and cryptography. Therefore, in this paper, the critical path delay and area of modulo adder are analyzed. An optimized diminished-one modulo adder for $2^{n}+1$ is proposed based on the analysis results.

• Theoretical comparison shows that the suggested modulo adder involves 23.41 % less area (transistors count) and 31.64 % less delay than the best existing design for an average bit-width.
• Synthesis result reveals that the proposed modulo adder involves 13.71 % less area and 14.5 % less delay compared to the best existing modulo adder structure design in the literature for an average bit-width.
• To observe the overall efficacy of the suggested modulo adder design, the area delay product (ADP) and power delay product (PDP) values of the proposed and existing modulo adder designs are computed using synthesis data. The values obtained for ADP and PDP reveal that the proposed design achieves a 26.2 % reduction in ADP and a 32.8 % improvement in PDP compared to the best available modulo-adder structure.

## Graphical abstract

**Figure:**
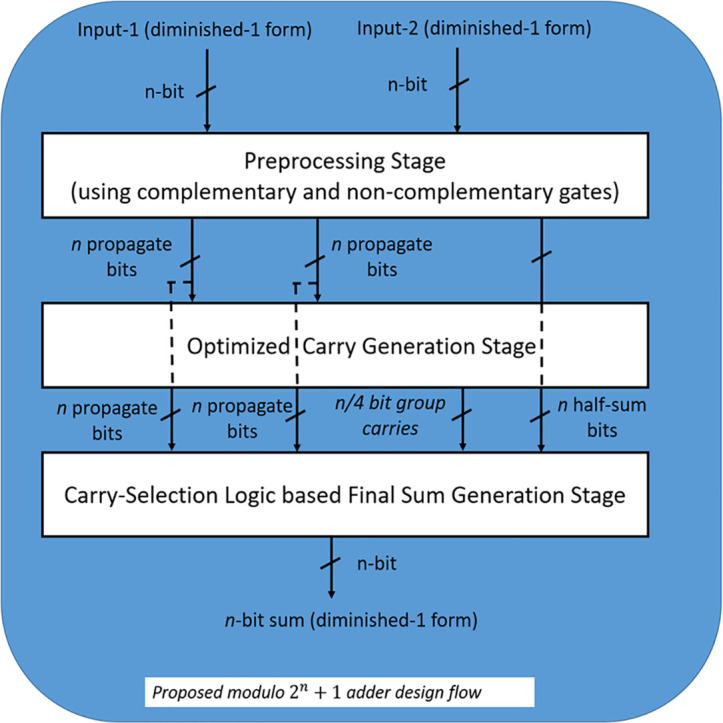

Specifications table

**Table:** 

Subject area:	VLSI Architecture Design
More specific subject area:	Modulo Adder
Name of your method:	Diminished-1 modulo adder
Name and reference of original method:	NA
Resource availability:	Synopsys Design Compiler

## Introduction

The Residue Number System (RNS) is extensively utilized in a wide range of DSP applications like finite impulse response filters (FIRs), digital signal processors, image processing modules, discrete cosine transform, cryptography, etc. [[1], [2], [3], [4]], to speed up the arithmetic operations. In RNS, a number is divided into residues (parts). It performs various arithmetic operations (such as addition, subtraction, multiplication etc.) in parallel for each part to reduce the computation time of the operations. The moduli set $\left\{2^{n}-1,\;2^{n},2^{n}+1\right\}$ is mostly used in RNS based systems. In this moduli set, more attention has been paid to modulo$\;2^{n}+1$ arithmetic operation because it works on n+1 bits binary operands, increasing delay and logic resources. To accommodate n+1 bits binary operand into n bits, diminished-1 representation has been suggested in [5], which reduces the delay and area of the modulo $2^{n}+1$ operations. Various modulo $2^{n}+1$ arithmetic operations, such as addition, subtraction and multiplication etc., are performed in many applications. In particular, modulo $2^{n}+1$ addition is the most commonly used operation in RNS- based systems. An efficient modulo $2^{n}+1$ adder design could be crucial in developing VLSI systems optimized for both area delay and power efficiency. Consequently, numerous architectures have been proposed in the literature to efficiently implement of $2^{n}+1$ adders. The chronological developments of these architectures are briefly discussed in the next paragraph.

Zimmermann et al. have suggested efficient VLSI architecture for $2^{n}+1$ modulo adder in which parallel prefix adder with end-around-carry is used to realize arithmetic operations [6]. In [7], authors have developed diminished-1 modulo $2^{n}+1$ adder architectures using two approaches, namely carry look-ahead and parallel prefix for its implementation. Subsequently, select-parallel-prefix addition block based diminished-1 modulo $2^{n}+1$ adder has been given in [8], which offers a good trade-off between area and speed. Further, Costas et al. [9] presented the area-delay efficient modulo $2^{n}+1$ adder using modified parallel-prefix carry computation and fast carry-increment stage. In [10], researchers have proposed a circular-carry-selection method to design diminished-1 modulo $2^{n}+1$ adder to minimize the area-delay and power-delay complexities. Subsequently, Vergos et al. [11] have developed modulo $2^{n}+1$ adder, where operands are represented in weighted form. Later on, diminished-1 modulo $2^{n}+1$ adder has been suggested in [12] in which authors have introduced new carry look-ahead and parallel-prefix adder structures for the fast implementation of carry computations.

In addition, the sparse carry computation-based efficient modulo $2^{n}+1$ adder architectures have been suggested in [[13], [14], [15]], where only group carries are calculated for the modulo addition. In [16], authors have proposed FFPGA-targeted modulo $2^{n}+1$ adder architectures with fast carry chains to speed up the arithmetic operations. Subsequently, delay efficient modulo $2^{n}+1$ adder has been realized in [17] using redundant carry-save forms of the operands. The $2^{n}+1$ modulo add-multiply component was developed in [18], which offers saving in area and delay compared to using individual adder and multiplier components. Further, Khan et al. [19] have given a comparative analysis of modulo adders using different parallel prefix adder architectures. In [20], authors have suggested modulo-generic adder circuits in which they have maximized sharing of components by merging two adders to optimize cell interconnect overhead. The carry skip logic-based modulo 2^n^ + 1 adder design has been suggested in [21] to achieve area-power efficiency. In [22], authors have recently analyzed diminished-1 modulo 2^n^ + 1 adder and subtractor circuits and suggested improved designs with less area and power consumption.

Further, a modified parallel prefix adder-based diminished-1 modulo $2^{n}+1$ adder design has been reported in [23]. This design uses a group-carry selection approach to optimize area and power consumption. From the literature, it is observed that most of the researchers have focused on reducing the area and delay complexities of modulo adder by removing the redundant logic present in the design. However, the logic redundancy in any modulo adder design is limited; therefore, it needs to explore another way to obtain area delay efficient modulo adder design. On the other hand, the majority of digital systems are implemented using complementary metal–oxide–semiconductor (CMOS) technology. Based on the literature, we have set certain objectives for this paper, which are stated below:

• To explore the CMOS library to find the possibilities of area-delay optimization of the modulo-adder design.
• To explore the logic redundancy (if any) available in the existing modulo-adder designs to minimize the area and power and improve the speed.

From the CMOS library, it is observed that the delay and area of complementary gates are less than that of non-complementary gates. These observations propose an efficient area delay diminished-1 modulo $2^{n}+1$ adder structure. The remaining portion of the article is presented under the section's method details, method validation, limitations, and conclusion.

## Method details

### Proposed structure for modulo adder

**Fig. 1:**
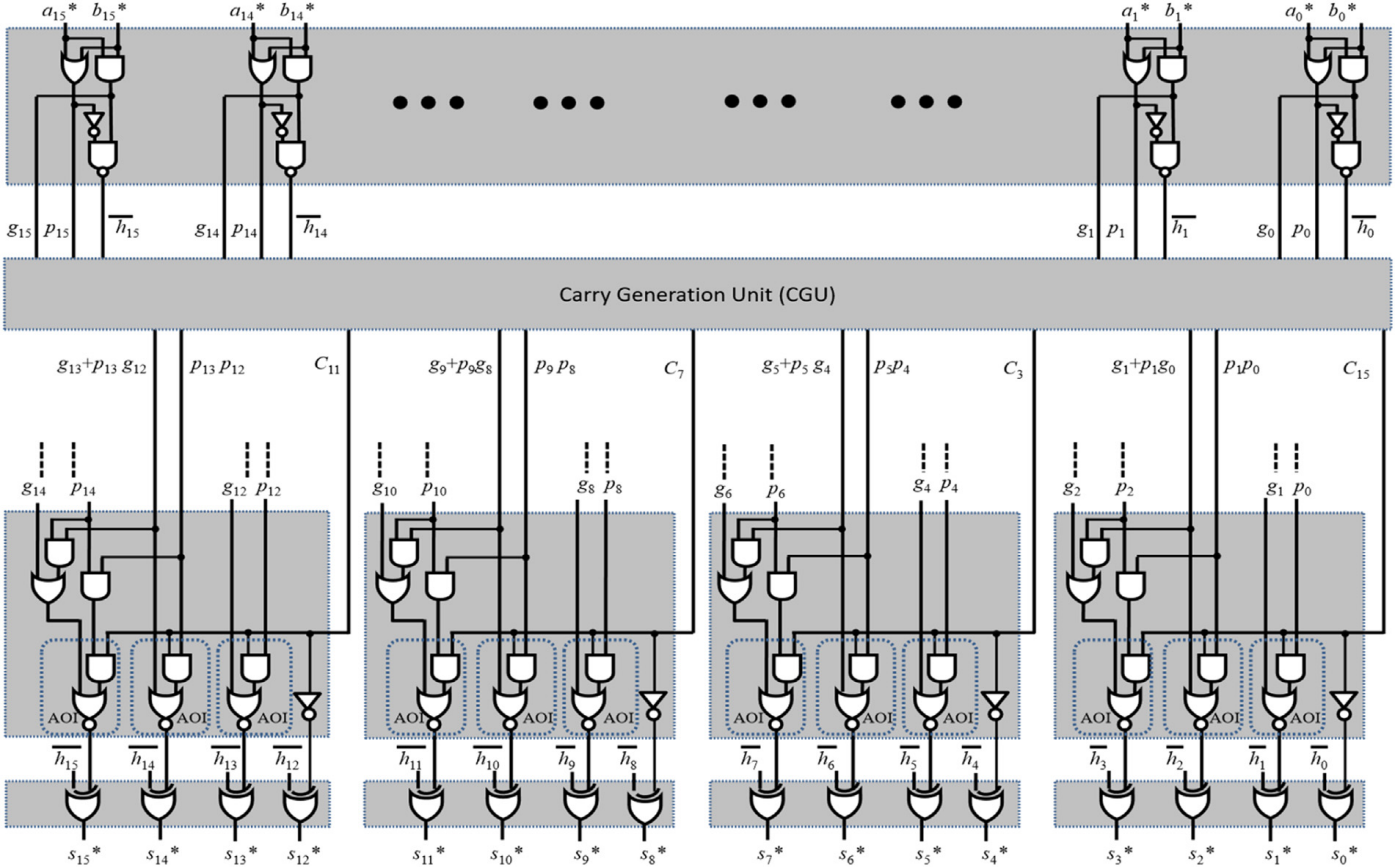
Proposed diminished-1 modulo $2^{n}+1$ adder.

The proposed diminished-1 module $2^{n}+1$ adder structure is shown in Fig. 1. It performs modulo addition in three stages. The first stage is a pre-processing stage that receives two diminished-1 numbers as input and produces propagate (p_i_), generate(g_i_), and half-sum bit (in complemented form, i.e., h_i_). The second stage is carry-generation, where all the intermediate group-carries are generated using g_i_ and p_i_ bits. Finally, the third stage is a sum-generation stage in which all the intermediate group carries are combined with the half-sum bits generated in the first stage and produce final sum bits. The detailed design strategies of each stage are explained in subsequent subsections.

### Pre-processing stage

Conventionally, in the pre-processing stage, three logic gates (XOR, AND and OR) are used to generate g_i_, p_i_ and h_i_ bits, where 0≤i≤n−1. In the proposed design, for the reduction of the delay in the critical path of the carry-generation as well as sum-generation stages, we have designed the pre-processing stage with maximal possible utilization of complementary logic cells. These half-sum bits have to be generated in complementary form, as shown in the pre-processing block of Fig. 1.

### Carry generation stage

**Fig. 2:**
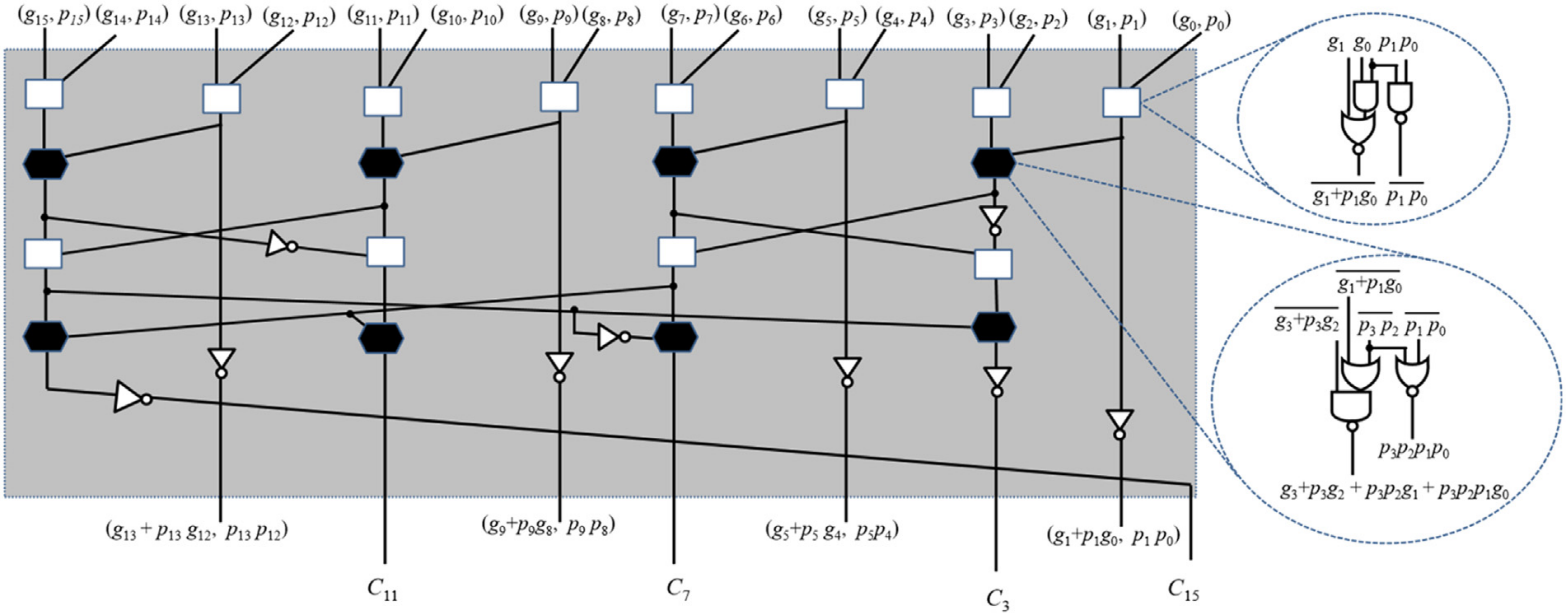
Proposed carry generation unit.

Generally, the carry generation stage involves $\log_{2}n$ levels of parallel-prefix logic cells (PPLCs) to generate the intermediate carries. The PPLC comprises of two AND and one OR gate. The critical path delay of this cell includes one AND and one OR gates. Therefore, the total delay of carry generation unit is $\log_{2}n\times \left(T_{A}+T_{O}\right)$, where $T_{A}\;\text{and}\;T_{O}$ are the delay of 2-input AND and OR gates, respectively. This type of carry generation stage involves more delay and requires more area. However, in the literature, the group carry selection-based carry generation stage has recently been utilized to reduce the area by minimizing the number of PLCs. But, to reduce delay, the optimization of PPLC is required. Therefore, we have gone through the Taiwan semiconductor manufacturing company (TSMC) 65 nm CMOS library for logic gates and observed that the delay of complementary gates AND-OR-Inverter (AOI) and OR-AND-Inverter (OAI) is less over complex gates (AND-OR and OR-AND). Based on this study, the complementary gates are utilized for the development of PPLCs with less delay. Apart from this, it is also observed that these complementary gates-based PPLCs give outputs in complementary forms, which is unsuitable for directly replacing the conventional PPLCs in the carry computation unit. Therefore, the existing carry computation unit needs to be modified so that complementary PPLCs are accommodated within it to reduce delay. Based on these modifications, two new PPLCs and modified carry computing structures are proposed, as shown in Fig. 2.

### Sum generation stage

In the sum generation stage, all the intermediate carries required for the final sum are calculated using group carries generated in the second stage. Later, these intermediate carries and half sum bits (generated in the first stage) are used to generate final sum bits of modulo addition. In the existing design, the sum generation unit is developed using AND, OR, AND-OR (AO), OR-AND (OA), and XOR gates, which consumes more area and delay. As discussed in the previous subsection, complementary gates are more area- and delay-efficient than non-complementary complex gates (AO and OA). Therefore, an existing sum-generation unit is analyzed to optimize area and delay. This analysis reveals that the complementary complex gates can be utilized only when the structure is redesigned. Based on these observations, the complementary gates (as much as possible) based sum generation circuit is derived and shown in Fig. 2.

### Theoretical comparison

The theoretical comparison is discussed in this section to observe the efficacy of the proposed modulo-adder design. Area (in the form of transistor counts) and delay (in ps) of different logic gates are extracted from the TSMC 65 nm library [24] and supplied in Table 1 for the theoretical comparison of the proposed design with the state-of-the-art designs. The area and delay of the suggested and current designs are computed and shown in Table 2 using Table 1. It is evident from Table 2 that the suggested modulo adder involves 47.62 %, 35.86 %, and 23.41 % less area (transistors count) compared to the Kogg-Stone parallel prefix adder (PPA) based design of [22], Skansky PPA-based design of [22] and design of [23] respectively, on an average bit-width. Similarly, the Proposed design takes 25.62 %, 25.62 %, and 31.64 % less delay in comparison to the Kogg-Stone PPA-based design of [22], Skansky PPA-based design of [22] and design of [23], respectively, on an average bit-width. The following section presents synthesis results and a discussion to verify the theoretical findings.

**Table 1:** Area and delay of the CMOS logic cells (TCBN65GPLUS TSMC 65 nm core library data-book.

Gates	Cell-Name	Delay (ps)	Are (Transistor Count)
AND	AN2D0	26.15	6
OR	OR2D0	28.5	6
NOR	NR2D0	13.95	4
NAND	ND2D0	12.75	4
NOT	INVD0	9.4	2
XOR	XOR2D0	42	12
AOI	AOI21D0	18.65	6
AO	AO21D0	33.45	8
OAI	OAI21D0	17.55	6

**Table 2:** Comparison of theoretical results for modulo adders.

Adder Design	bit-width (m)	Area (Transistors)	Delay (ps)
Design based on Kogg-Stone PPA [22]	16 32	1462 3350	235.4 268.85
Design based on Sklansky PPA [22]	16 32	1224 2664	235.4 268.85
Design of [23]	16 32	1042 2194	254.2 287.65
Proposed	16 32	802 1672	178 196.65

Energy=Delay×Power, Excess energy over the proposed modulo adder.

### Method validation

The proposed and existing modulo-adder designs are coded in VHDL for bit-width 16 and 32 for the method validation. The intended and contemporary modulo adder designs are coded in VHDL for bit-width 16 and 32 to enable experimental investigation. Furthermore, using TSMC 65 nm CMOS Library cells, these designs are synthesized in Synopsys Design Compiler. Table 3 lists the reported values for area, delay, and power. From the synthesis results given in Table 3, it can be seen that the proposed modulo adder involves 34.64 %, 23.21 %, and 13.71 % less area compared to the Kogg-Stone PPA-based design of [22], Skansky PPA-based design of [22] and design of [23] respectively, on an average bit-width. Similarly, the Proposed design takes 12.05 %, 11.14 %, and 14.5 % less delay in comparison to the Kogg-Stone PPA-based design of [22], Sklansky PPA-based design of [22], and design of [23], respectively, on an average bit-width. It is clear from the comparison analysis that the suggested designs are more efficient in terms of area, delay, and power than existing designs, which is also supported by the theoretical comparison covered in the preceding section.

**Table 3:** Comparison of synthesis results for intended and contemporary modulo adders.

Modulo Adder	Width (m)	Delay (ns)	Area (µm2)	Power (µW)
Design based on Kogg-Stone PPA [22]	16 32	0.49 0.59	586.8 1252.0	212.85 374.30
Design based on Sklansky PPA [22]	16 32	0.48 0.59	489.6 1088.5	172.60 317.76
Design of [23]	16 32	0.51 0.60	444.3 948.4	175.47 317.83
Proposed	16 32	0.43 0.52	393.8 796.3	144.13 238.31

**Fig. 3:**
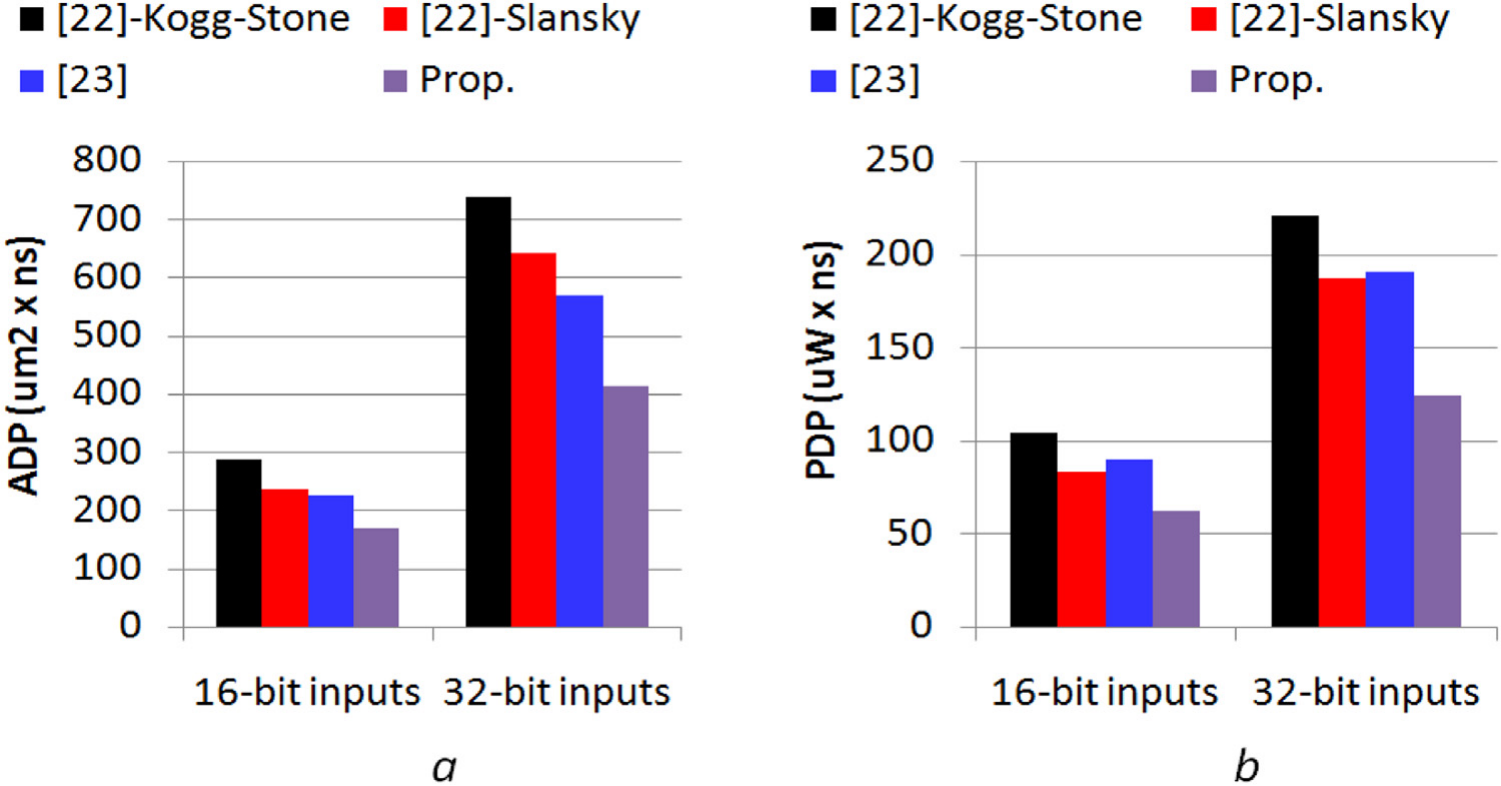
Comparison of (a) ADP and (b) PDP.

The area-delay-product (ADP=Area×Delay) and power-delay-product (PDP=Power×Delay) values are computed using the synthesis data provided in Table 3 to observe the overall efficacy of the suggested modulo adder design. These calculated values of ADP and PDP are plotted in the form of bar-graph shown in Fig. 3(a and b) for comparison. From the bar graph, it can be seen that the proposed modulo adder design gives an improvement in ADP and PDP by 42.52 % and 42.23 % over the Kogg-Stone PPA-based design of [22]; 31.73 % and 29.54 % over Sklansky PPA based design of [22]; 26.24 % and 32.88 % over [23], respectively. Consequently, it is evident from the explanation above that the suggested modulo adder is more efficient and can be utilized to create effective VLSI digital systems.

### Limitations

The limitation of the proposed design is that the bit-width (n) should be in the order of $2^{n}+1$ to get maximum efficiency in terms of area, delay, and power.

## Conclusion

Modulo adder is a widely used arithmetic component in many DSP applications, such as FIR, IIR filters, digital signal processors, image processing modules, discrete cosine transform, and cryptography. Therefore, in this paper, the CMOS library and logic redundancy available in the existing designs were explored to find the possibilities of area delay optimization of the modulo-adder design. From the CMOS library, it is observed that the delay and area of complementary gates are less than that of complex gates. These observations propose area delay efficient diminished-1 modulo 2 to the n plus 1adder structure. The theoretical comparison shows that the suggested modulo adder involves 23.41 % less area (transistor count) and 31.64 % less delay than the best existing design for an average bit-width. Synthesis results reveal that the proposed modulo adder involves 13.71 % less area and 14.5 % less delay compared to the best available existing modulo adder structure design in the literature for an average bit-width, which validates the theoretical comparison achievements of the proposed design. Overall, the values obtained for ADP and PDP reveal that the proposed design achieves a 26.2 % reduction in ADP and a 32.8 % improvement in PDP compared to the best available modulo-adder structure. Thus, the proposed modulo-adder design is superior for developing area- and delay-efficient VLSI digital systems. In the future, this design can be explored to develop the area-delay efficient modulo multiplier structure.

## Ethics statements

This research did not involve research on humans or animals, and no data is involved from social media platforms.

## CRediT authorship contribution statement

**Subodh Kumar Singhal:** Validation, Visualization. **Sumit Kumar:** Funding acquisition, Validation, Methodology, Writing – original draft. **Sujit Kumar Patel:** Conceptualization. **K. Anjali Rao:** Supervision, Writing – review & editing. **Gaurav Saxena:** Writing – original draft.

## Declaration of competing interests

The authors declare that they have no known competing financial interests or personal relationships that could have appeared to influence the work reported in this paper.

## Data Availability

No data was used for the research described in the article.
